# Robo 4 Counteracts Angiogenesis in Herpetic Stromal Keratitis

**DOI:** 10.1371/journal.pone.0141925

**Published:** 2015-12-31

**Authors:** Fernanda Gimenez, Sachin Mulik, Tamara Veiga-Parga, Siddheshvar Bhela, Barry T. Rouse

**Affiliations:** 1 Department of Biomedical and Diagnostic Sciences, College of Veterinary Medicine, University of Tennessee, 1414 Cumberland Avenue, Knoxville, TN, 37996, United States of America; 2 Immune Disease Institute and Program in Cellular and Molecular Medicine, Children’s Hospital Boston, Harvard Medical School, Boston, MA, United States of America; University of Georgia, UNITED STATES

## Abstract

The cornea is a complex tissue that must preserve its transparency to maintain optimal vision. However, in some circumstances, damage to the eye can result in neovascularization that impairs vision. This outcome can occur when herpes simplex virus type 1 (HSV-1) causes the immunoinflammatory lesion stromal keratitis (SK). Potentially useful measures to control the severity of SK are to target angiogenesis which with herpetic SK invariably involves VEGF. One such way to control angiogenesis involves the endothelial receptor Robo4 (R4), which upon interaction with another protein activates an antiangiogenic pathway that counteracts VEGF downstream signaling. In this study we show that mice unable to produce R4 because of gene knockout developed significantly higher angiogenesis after HSV-1 ocular infection than did infected wild type (WT) controls. Moreover, providing additional soluble R4 (sR4) protein by subconjunctival administration to R4 KO HSV-1 infected mice substantially rescued the WT phenotype. Finally, administration of sR4 to WT HSV-1 infected mice diminished the extent of corneal angiogenesis compared to WT control animals. Our results indicate that sR4 could represent a useful therapeutic tool to counteract corneal angiogenesis and help control the severity of SK.

## Introduction

The cornea needs to be transparent to allow transmission of incident light so as to achieve optimal vision. While the cornea has different mechanisms to maintain its transparency, certain injuries can result in corneal opacification and impaired vision [1,2]. Such is the case with stromal keratitis (SK), a lesion that can follow corneal infection with herpes simplex virus (HSV-1), which in humans is usually the consequence of repeated viral reactivation of latent infection in the peripheral nervous system [3]. SK involves multiple events one of which is the formation of new blood vessels into the normally avascular cornea. Accordingly, diminishing the extent of corneal neovascularization (CV) represents a useful approach to therapy[4]. The main target so far investigated has been the principal stimulus for angiogenesis, vascular endothelial growth factor (VEGF) and its receptors. These treatment approaches have included the use of recombinant soluble VEGF receptor (VEGF-R1), a fusion protein also called the VEGF trap [5]; recombinant humanized monoclonal antibody known as Bevacizumab [6]; VEGF and VEGF receptor silencing RNAs [7]; SRC kinase inhibitors [6] and the inhibition of some miRNAs [8]. An alternative approach that could control CV, is to exploit the mechanisms the host itself has to limit the extent of VEGF induced angiogenesis. This mechanism uses the Robo4 (R4) receptor, a member of the axon guidance receptor family which is expressed on angiogenic endothelial cells [9,10]. Upon interaction with its ligand, R4 generates a negative signal in the cell that diminishes the VEGF response [11–14]. In support of this, when R4 is absent because of gene knockout, mice may develop accelerated angiogenesis in tissues such as the retina [11]. Currently any role for R4 at modulating HSV-1nduced angiogenesis in the cornea has not been reported.

In the present report, we evaluate the role of R4 signaling in an ocular model of CV and inflammation that follows primary ocular infection with HSV-1. We demonstrate that mice lacking R4, because of gene knockout, develop more severe corneal angiogenesis compared to WT mice. Moreover, administration of soluble R4 (sR4) to R4 KO HSV-1 infected mice substantially rescued the WT phenotype. In addition, provision of sR4 by subconjunctival administration to WT infected animals significantly diminished the extent of corneal angiogenesis. It is possible that the outcome observed in R4 KO mice, was due to the interaction of sR4 with the vascular specific axon receptor uncoordinated homolog 5β (UNC5β), however further research is needed to verify this issue. Since the results suggest that the administration of sR4 promotes the activation of an antiangiogenic pathway, this approach may represent a valuable therapeutic tool to control corneal angiogenesis related to HSV-1 induced SK.

## Materials and Methods

### Mice

Female 6–8 wks old C57BL/6 mice were purchased from Harlan Sprague Dawley Inc. (Indianapolis, Indiana, USA). Robo4 knockout (R4 KO) mice were the kind gift of Christopher A Jones (University of Utah). The animals were housed in American Association of Laboratory Animal Care-approved facilities at the University of Tennessee, Knoxville. All investigations followed guidelines of the institutional animal care and use committee.

### Ethics Statement

This study was carried out in strict accordance with the recommendations in the Guide for the Care and Use of Laboratory Animals of the National Institutes of Health. The protocol was approved by the University of Tennessee Animal Care and Use committee (protocol approval numbers 1253–0412 and 1244–0412). All procedures were performed under tribromoethanol (avertin) anesthesia, and all efforts were made to minimize suffering.

### Virus

HSV-1 strain RE Tumpey was propagated in Vero cell monolayers (ATCC no: CCL81). Virus was grown in Vero cell monolayers (American Type Culture Collection, Manassas, VA), titrated, and stored in aliquots at –80°C until used.

### Corneal HSV-1 infection and scoring

Corneal infections of mice were performed under deep anesthesia. The mice were lightly scarified on their corneas with a 27-gauge needle and a 3μl drop containing HSV-1 RE was applied to one eye. When experiments included R4 KO mice the animals were infected with 8 x 10^3^ mice PFU of HSV-1. When experiments included only WT mice, animals were infected with 10^4^ PFU of HSV-1. The SK lesion severity and angiogenesis in the eyes of mice were examined by slit-lamp biomicroscopy (Kowa Company, Nagoya, Japan). The scoring system was as follows: 0, normal cornea; +1, mild corneal haze; +2, moderate corneal opacity or scarring; +3, severe corneal opacity but iris visible; +4, opaque cornea and corneal ulcer; +5, corneal rupture and necrotizing keratitis [5]. The severity of angiogenesis was recorded as described previously [15]. According to this system, a grade of 4 for a given quadrant of the circle represents a centripetal growth of 1.5 mm toward the corneal center. The score of the four quadrants of the eye were then summed to derive the neovessel index (range 0–16) for each eye at a given time point.

### Subconjunctival Injections

Subconjunctival injections were performed as previously reported [16]. Briefly, these injections were performed using a 2-cm, 32-gauge needle and syringe (Hamilton, Reno, NV) to penetrate into the subconjunctival space.

### Murine Treatment with soluble Robo4 (sR4)

WT and R4 KO mice were ocularly infected with HSV-1 RE Tumpey. WT mice were treated with PBS vehicle (WT control). R4 KO mice were separated into two groups, one of which was treated with sR4 (R4 KO treated) and the other with vehicle (R4 KO control). sR4 was kindly donated by Ryan Watts (Genentech Inc.). The treatment started 2 days pi., with additional daily doses until day 14 pi. In another experiments WT mice were infected with HSV-1 RE Tumpey and were divided in two groups, one of which received vehicle (control) (PBS) and the other one sR4 (treated). Both treatments were administered daily from day 2 pi. to day 14 pi. These animals were carefully followed for the progression of angiogenesis and SK development.

### Flow Cytometry

Corneal single cell suspensions were prepared following Liberase digestion of corneas collected at day 15 pi. Aliquots of the above single-cell suspensions were stained for CD4-FITC, CD45-PerCP, CD31-allophycocyanin, CD11b-PE and Ly6G-Pacific blue cell surface markers (All from BD Biosciences Pharmigen) for 30 minutes on ice. Thereafter, cells were washed twice and resuspended in 1% para-formaldehyde. The stained samples were acquired FACS LSR (BD Biosciences) and the data were analyzed using the FlowJo software.

### Quantification of mRNA expression levels by quantitative real time PCR (Q-RT-PCR)

Total mRNA was isolated from corneal cells using TRIzol LS reagent (Invitrogen). For RNA, cDNA was made with 500 ng of RNA using oligo (dT) primer and ImProm-II^™^ Reverse Transcription System (Promega). TaqMan gene expression assays (IL-6, IL-1β, CXCL-1, VEGF) were purchased from Applied Biosystems and were used to quantify mRNAs using a 7500 Fast Real-Time PCR System (Applied Biosystems). The expression levels of the target genes were normalized to β-actin and with the ΔCT method, and relative quantification between control and infected mice was performed using the (2^-ΔΔCT^) *1000 formula.

### Western Blot Analysis

The corneal cells were lysed and total protein in the supernatants was quantified using BCA protein assay kit (Thermo scientific, Waltman, MA). Samples were denatured in Laemmli buffer and resolved by SDS-PAGE and proteins were transferred onto a PVDF membrane. The membrane was blocked with 5% milk in Tris-buffered saline with Tween 20 at room temperature for 1 hour and subjected to incubation with specific primary and secondary antibodies. Proteins bands were visualized using chemiluminiscent HRP substrate (Millipore, Billerica, MA). After keeping in stripping buffer for 10 minutes, the membrane was re-probed using anti β-actin antibody. The antibodies used were as follows: anti Phospho-Src (Tyr416), anti Src (C-20), anti β-actin (AC 74). Protein concentration was determined relative to β actin and quantified using Image J software.

### Statistics

The statistical significance between two groups was determined using unpaired one-tailed student's t test. When data did not show normal distribution, the Matt- Whitney test was used. One-way ANOVA with Tukey’s multiple comparison tests was used to calculate the level of significance of the experiments with more than two groups to compare. When P ≤ 0.001 (***), P ≤ 0.01 (**), P ≤ 0.05 (*) were considered as significant and results were expressed as mean ± SEM. For all statistical analysis, GraphPad Prism software was used.

## Results

### Inhibition of R4 pathway increases angiogenesis and SK lesions

**Fig 1:**
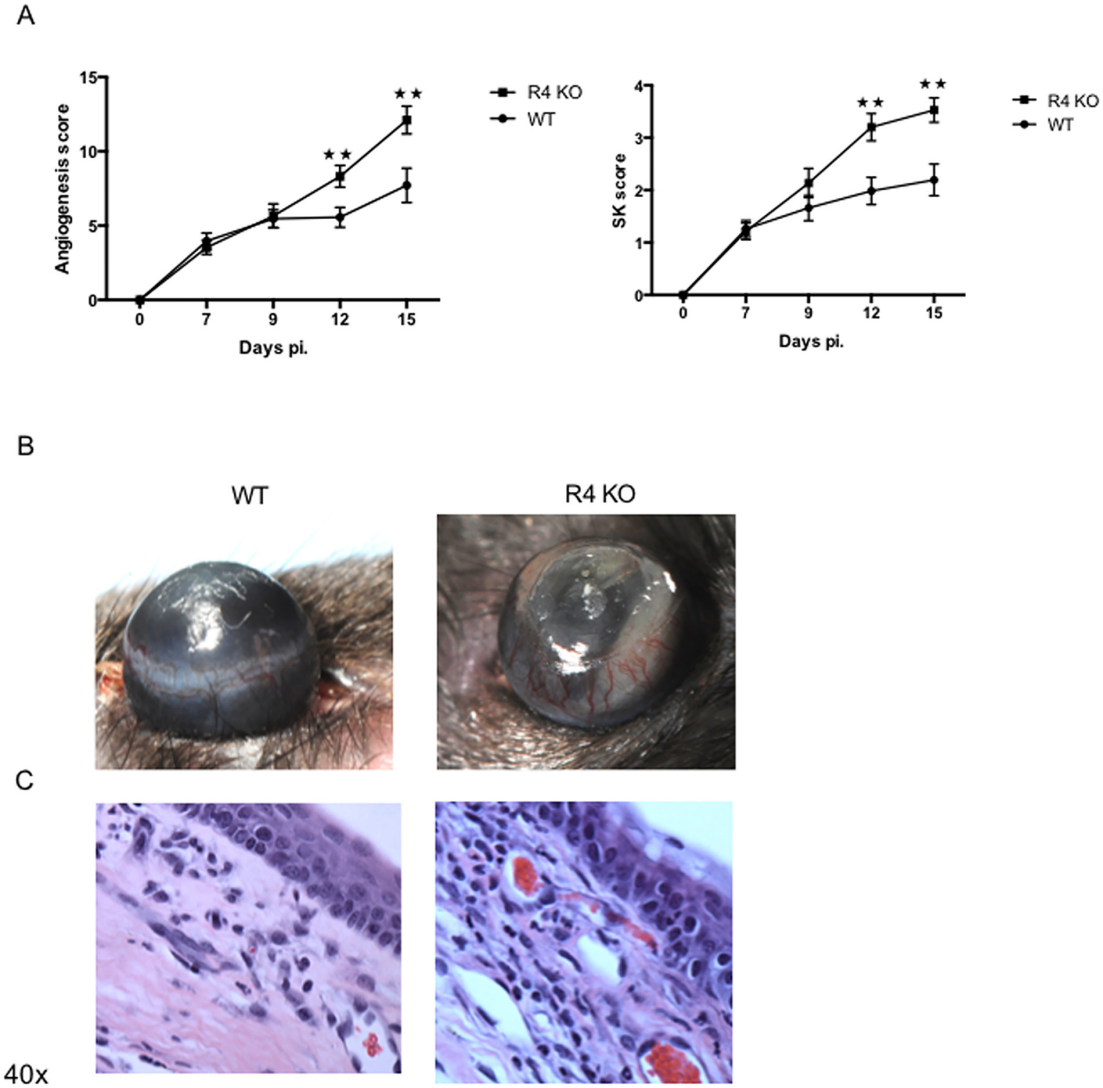
Robo4 deficient mice are more susceptible to HSV-1 infection. R4 KO mice were infected with HSV-1 RE. (A) SK lesion and angiogenesis severity were significantly increased in R4 KO mice compared to WT mice on day 15 pi. (B) Representative eye photos show increased SK lesion and angiogenesis severity in R4 KO compared to WT mice (C) Eyes were processed for cryo-sections on day 15 pi. Hematoxilyn and eosin staining was carried out on 6 μm sections, and pictures were taken at 40x magnification. The sections show decreased cellular infiltration in R4 KO mice compared to WT mice. Data are representative of three independent experiments and show mean values ± SEM (*n* = 15 mice/group). ***p ≤ 0.001, **p ≤ 0.01, *p ≤ 0.05. Statistical levels of significance were analyzed by *t* test.

**Fig 2:**
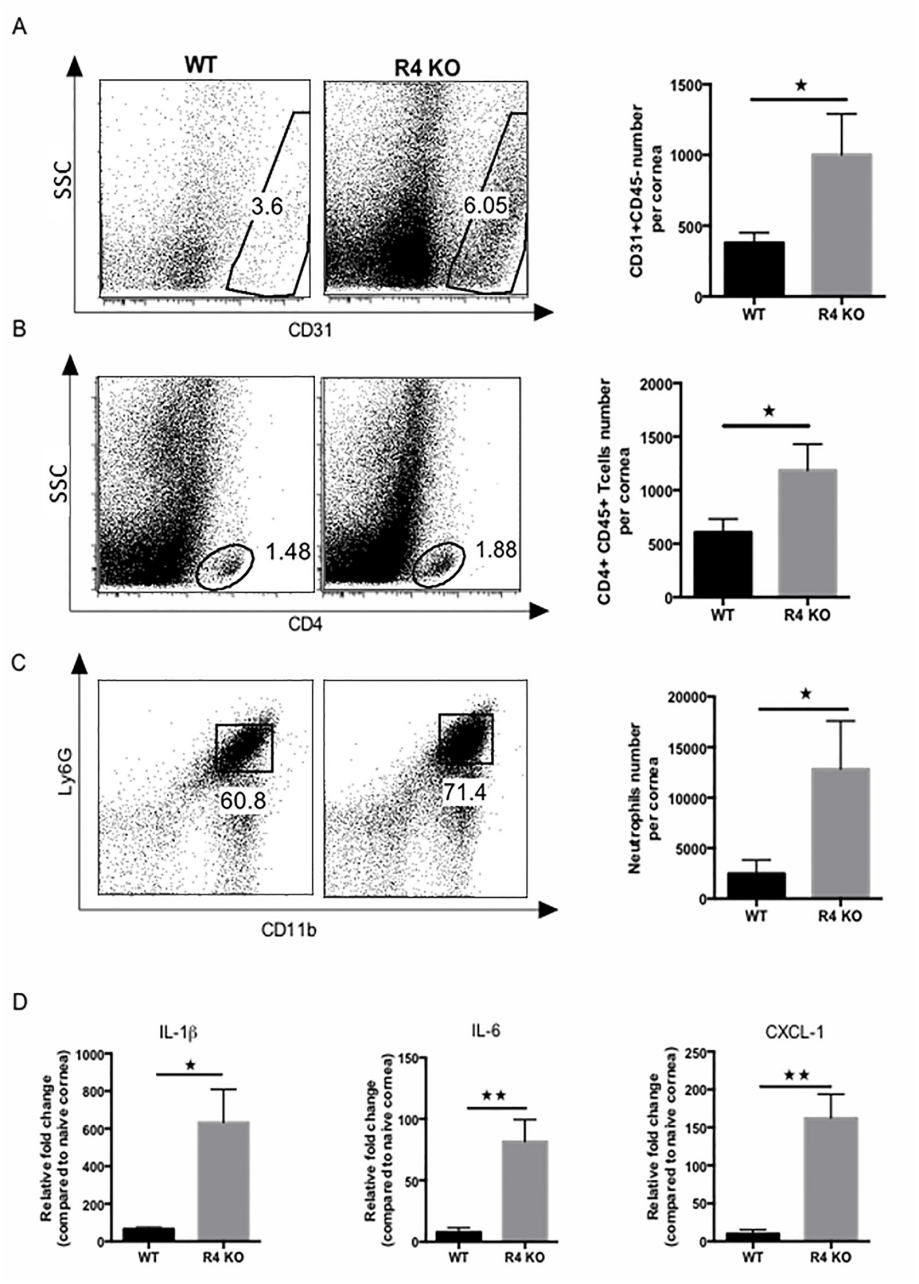
Robo4 deficient mice present more inflammation and vascularization. Robo4 knockout (R4 KO) and WT mice were infected with HSV-1 RE and at day 15 pi. corneas were collected and pooled for analysis by flow cytometry or Q-RT-PCR. The frequency and total cell number per cornea for (A) endothelial cells (CD31+) gated on total CD45- cells infiltrate, (B) CD4+ T cells (CD4+) (gated on total CD45+ cells infiltrate) and (C) neutrophils (Ly6G+ CD11b+ gated on total CD45+ cells infiltrate) show significant increase in R4 KO mice. Data are a combination of 3 independent experiments and show mean values ± SEM (n = 7 and each sample is representative of 2 corneas). ***p ≤ 0.001, **p ≤ 0.01, *p ≤ 0.05. Statistical levels of significance were analyzed by *t* test. (E) Relative fold change in mRNA expression of IL-1β, IL-6 and CXCL-1 was examined and compared between WT and Robo4 KO mice on day 15 pi. by Q-RT-PCR. Data represent means ± SEM from two different independent experiments (n = 3 and each sample is representative of 5 corneas). ***p ≤ 0.001, **p ≤ 0.01, *p ≤ 0.05. Statistical levels of significance were analyzed by *t* test.

To evaluate the role of R4 in HSV-1 induced SK, the outcome of infection was compared over a 15 day time period in ocularly infected R4 KO and WT mice. While in R4 KO mice SK lesions started to be evident from day 9 pi. onward as in WT mice, angiogenesis and SK scores were significantly higher in R4 KO mice and peaked at day 15 pi. (p = 0.001, for both scores) (Fig 1A and 1B). Examination of histological sections at day 15 pi. also showed increased lesion severity in the R4 KO animals compared to WT sections (Fig 1C). In independent experiments of the same design, corneal tissues were collected from both groups on day 15 pi. and collagen digested to recover cells for FACS analysis. The numbers of CD31+ corneal endothelial cells (blood vessels) and SK lesion inducing CD4+ T cells increased around 2 fold for both cell types (p = 0.04 and p = 0.02, respectively) (Fig 2A and 2B). Additionally neutrophils were increased by approximately 3 fold (p = 0.02) (Fig 2C). Finally, at the same time point, pools of corneas from WT and R4 KO mice were collected for the measurement by Q-RT-PCR of proinflammatory cytokines. These included IL-1β, IL-6, and CXCL-1 all of which were significantly increased in R4 KO compared to WT mice (p = 0.01, p = 0.002 and p = 0.04, respectively) (Fig 2D). In conclusion, these results show that R4 plays a role to limit the extent of angiogenesis that follows HSV-1 infection.

### Administration of sR4 protein reduces angiogenesis and SK score in R4 KO mice

**Fig 3:**
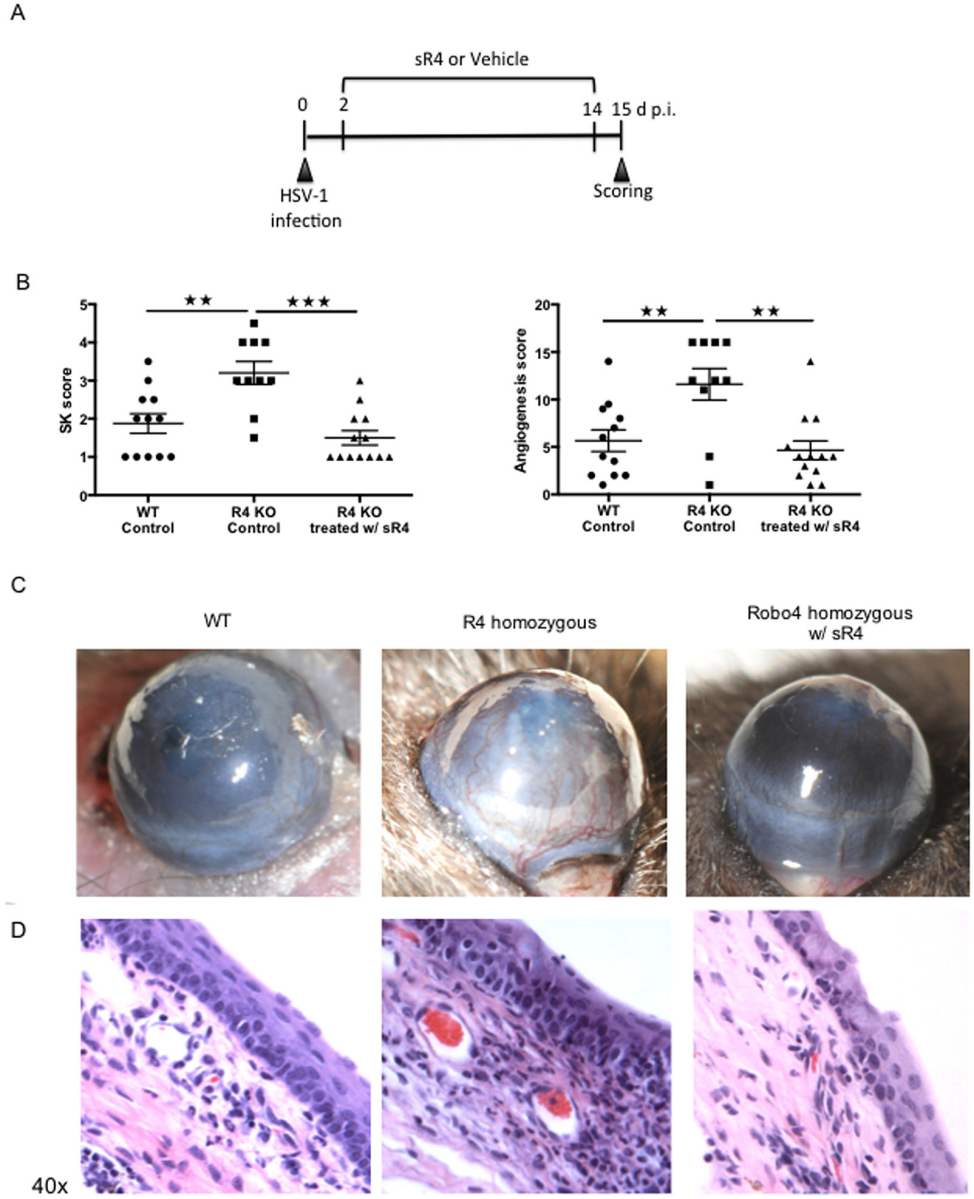
Administration of soluble Robo4 (sR4) shifts the Robo4 knockout to the WT phenotype. WT and Robo4 knockout mice (R4 KO) were infected with HSV-1 RE. (A) R4 KO mice received either sR4 (R4 KO treated w/ sR4) or vehicle (R4 KO Control) from 2 to 14 days pi. WT mice were included and received vehicle (WT Control) under the same regimen previously stated. (B) SK lesions and angiogenesis severity was decreased in R4 KO mice treated with sR4. (C) Representative eye photos show that R4 KO mice treated with sR4 do not develop the severe phenotype that R4 KO control animals do (D) Hematoxylin and eosin staining was carried out on 6-μm sections, and pictures were taken 40 x magnification. Representative eye sections show decreased cellular infiltration in R4 KO treated w/ sR4 and WT control compared to R4 KO control mice. Data are representative of two independent experiments and show mean values ± SEM (n = 12 mice/group). ***p ≤ 0.001,**p ≤ 0.01,*p ≤ 0.05. Statistical levels of significance were analyzed by one-way ANOVA test with Tuckey’s post hoc test settings.

To determine if the provision of the soluble extracellular domain of R4 could reduce the increased vascularization seen in R4 KO mice, WT and two groups of R4 KO animals were ocularly infected with HSV-1. At day 2 pi., while one group of R4 KO mice received daily administration of 10 μg sR4 until day 14 pi. (Fig 3A), the other two control groups (R4 KO control and WT control) received daily administration of PBS vehicle during the same time frame. Clinical evaluation revealed that compared to R4 KO animals, sR4 treated R4 KO mice developed reduced clinical angiogenesis and SK scores (p<0.05), declining to levels similar to that observed in WT animals (Fig 3B and 3C). The clinical observations were also supported by histopathology. Increased cellular infiltration was evident in R4 KO mice without treatment compared to WT and sR4 treated R4 KO animals (Fig 3D). Taken together, these results show that the administration of sR4 in the R4 KO acted to diminish the extent of lesions to those observed in WT animals, further demonstrating that Robo4 is a molecule that acts to constrain HSV-1 induced corneal angiogenesis.

### Administration of sR4 diminishes angiogenesis and SK

**Fig 4:**
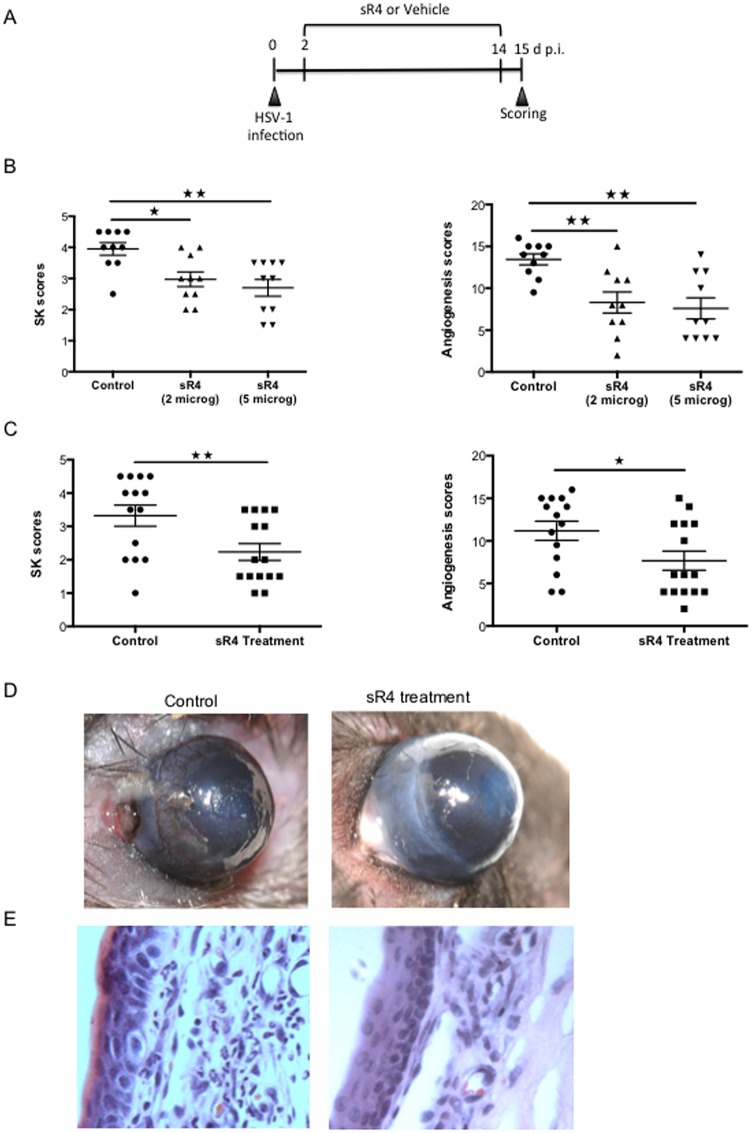
Preventive administration of soluble Robo4 (sR4) reduces lesion severity in HSV-1 infected mice. WT mice were infected with HSV-1 RE and treated with sR4 or vehicle (PBS) (A) The sR4 treatment was given to HSV-1 infected mice as shown (B) Dose dependent inhibition of angiogenesis scores after sR4 treatment. Data represents means ± SEM from three different experiments (n = 10) (C) sR4 treatment regimen resulted in SK and angiogenesis scores reduction. Data are representative of three independent experiments and show mean values ± SEM (*n* = 15 mice/group). ***p ≤ 0.001, **p ≤ 0.01, *p ≤ 0.05. Statistical levels of significance were analyzed by *t* test. (D) Representative eye photos show decreased SK lesion and angiogenesis severity in sR4 treated mice compared to control mice (E) Eyes were processed for cryo-sections on day 15 pi. Hematoxilyn and eosin staining was carried out on 6 μm sections, and pictures were taken at 40x magnification. The sections show decreased cellular infiltration in mice treated with sR4 compared to control mice.

**Fig 5:**
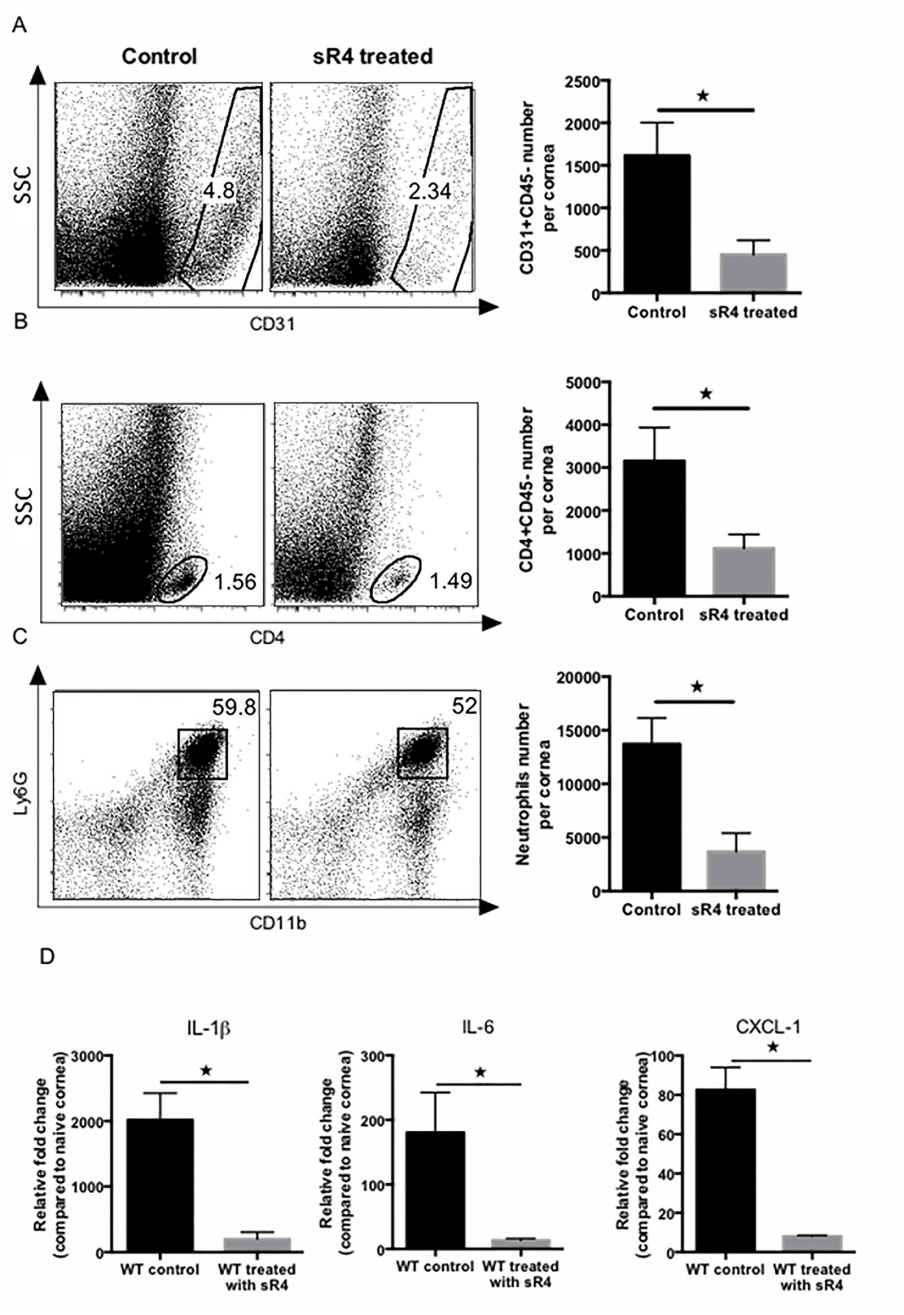
Provision of sR4 reduces corneal inflammation and angiogenesis. WT mice were infected with HSV-1 RE and treated with sR4 of vehicle (PBS) from day 2 pi. to 14 pi. At day 15 pi. corneas were collected and pooled for analysis by flow cytometry or Q-RT-PCR. The frequency and total cell number per cornea for (A) endothelial cells (CD31+) gated on total CD45- cells infiltrate, (B) CD4+ T cells (CD4+) (gated on total CD45+ cells infiltrate) and (C) neutrophils (Ly6G+ CD11b+ gated on total CD45+ cells infiltrate) show significant decrease in sR4 treated mice compared to WT mice. Data are a combination of 3 independent experiments and show mean values ± SEM (n = 7 and each sample is representative of 2 corneas). ***p ≤ 0.001, **p ≤ 0.01, *p ≤ 0.05. Statistical levels of significance were analyzed by *t* test. (D) Relative fold change in mRNA expression of IL-1β, IL-6 and CXCL-1 was examined and compared between sR4 treated and control mice on day 15 pi. by Q-RT-PCR. Data represent means ± SEM from three different independent experiments (n = 2 and each sample is representative of 6 corneas). ***p ≤ 0.001, **p ≤ 0.01, *p ≤ 0.05. Statistical levels of significance were analyzed by *t* test.

**Fig 6:**
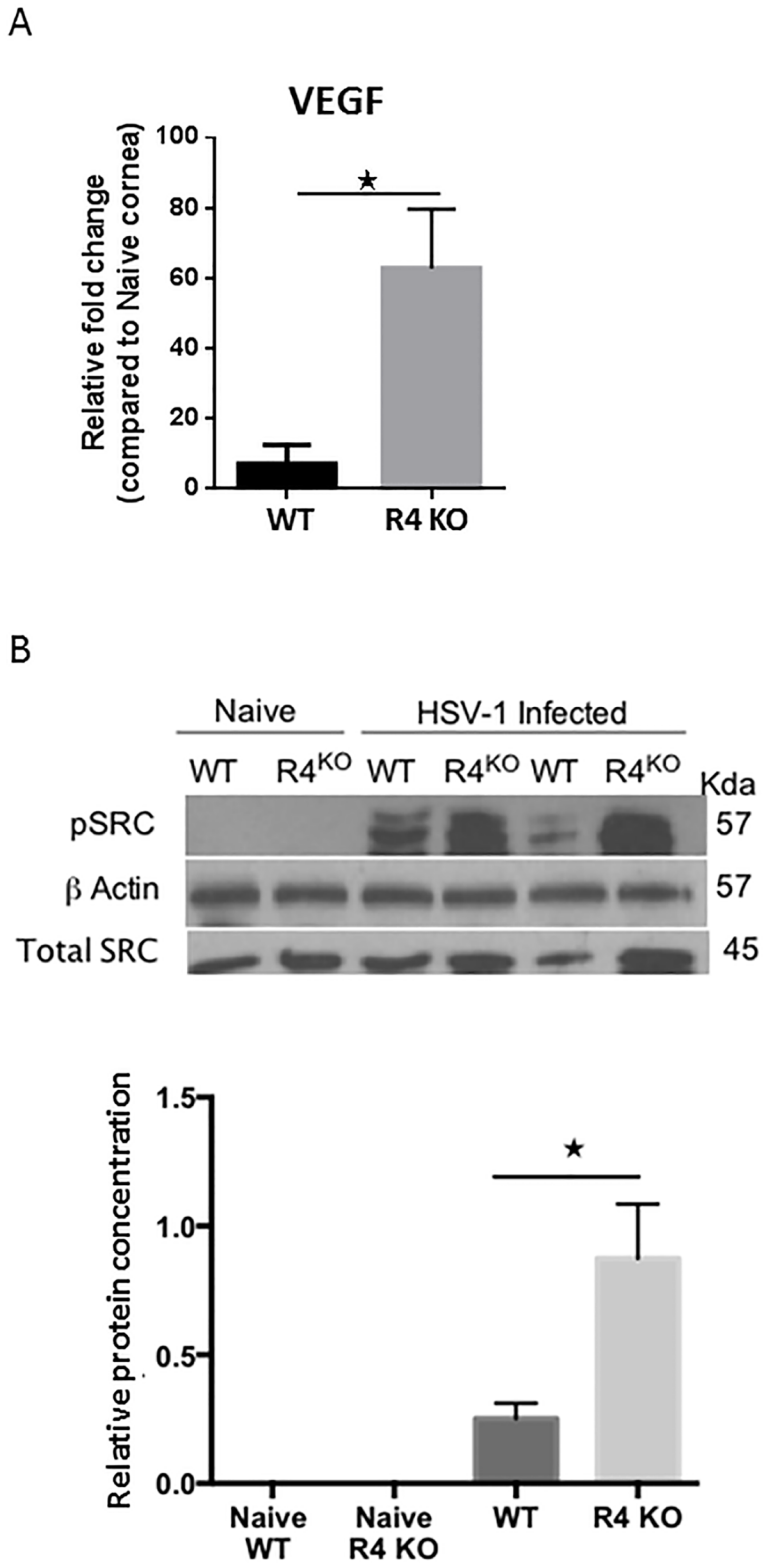
Increased lesion severity in Robo4 KO mice is due to higher VEGF levels and signaling. WT and R4 KO mice were infected with HSV-1 RE in one eye and corneas were collected and pooled for analysis by WB and by Q-RT-PCR. (A) Relative fold change in mRNA expression of VEGF was examined and compared between WT and R4 KO animals. Data represent means ± SEM (n = 2 and each sample is representative of 6 corneas). ***p ≤ 0.001, **p ≤ 0.01, *p ≤ 0.05. Statistical levels of significance were analyzed by *t* test. (B) WB analysis of corneal lysates. R4 KO animals have increased VEGF signaling showed by increased SRC phosphorylation at day 15 pi. (n = 3 and each sample is representative of 5 corneas).

In order to further evaluate the effect of sR4 in the progression of HSV-1-induced angiogenesis, WT animals were treated subconjunctivally with sR4 or vehicle starting on day 2 pi. and repeated daily until day 14 pi. (Fig 4A). As shown in Fig 4B, sR4 treatment caused reduced levels of angiogenesis and SK development with the maximum reduction observed at the higher dose used for treatment (SK score control:4 vs SK score treated: 2–3, angiogenesis score control:14 vs angiogenesis score control: to 8–10). That the sR4 treatment was effective at reducing lesions was also evident in the pictures and histological sections (Fig 4C, 4D and 4E). The extent of vascularization and inflammatory ocular reaction were compared by sacrificing treated and control animals at day 15 pi., following collagenase digestion and recovering corneal cells for FACS analysis. As is evident, the number of CD31^+^ endothelial cells were reduced around 3 fold in animals treated with sR4 compared to the controls (p = 0.02) (Fig 5A). In addition CD4^+^, and neutrophils were reduced around 3 and 2.5 fold respectively, (p = 0.02 and p = 0.01) in treated compared with control mice (Fig 5B and 5C). Finally, pools of 6 corneas HSV-1 infected sR4 treated and non treated were collected at day 15 pi. and processed to prepare RNA. As is evident in Fig 6D, animals treated with sR4 showed reduced of IL-1β, IL-6 and CXCL-1 transcripts compared with infected controls (p = 0.02, p = 0.01 and p = 0.01, respectively). Thus, taken together our results demonstrate that sR4 is a useful protein to modulate the extent of angiogenesis as well as the consequent immunopathology that follows HSV-1 infection.

### Increased angiogenesis in R4 KO mice is due to higher levels of VEGF and VEGF signaling

To explain the increased vascularization in R4 KO compared to WT mice, we measured VEGF levels and the extent of SRC phosphorylation (pSRC). Both groups of animals were ocularly infected with HSV-1 and the corneas were collected at day 15 pi. and analyzed by Western blotting. WT and R4 KO naïve corneas were used as controls. In additional experiments at the same time point, pools of corneas from WT and R4 KO mice were collected for the measurement of VEGF by Q-RT-PCR. The results showed that VEGF transcripts were significantly increased in R4 KO compared to WT mice (p = 0.04) (Fig 6A). Whereas pSRC was undetectable in naïve corneas of both groups, pSRC was significantly increased in R4 KO compared to WT infected corneal lysates (Fig 6B). The data indicate that absence of R4 in mice leads to increased levels of VEGF during SK and subsequently to heightened VEGF signaling.

## Discussion

This communication explores the role of the vascular molecule R4 in controlling the magnitude of the neovascular response to ocular infection with HSV-1. We demonstrate that R4 serves to limit the extent of new blood vessel development and the severity of SK lesions following HSV-1 infection. Evidence for such a function of R4 came from two sets of observations. Firstly animals unable to express R4 because of gene knockout developed earlier and more intense neovascular responses, and secondly the subconjunctival administration of a soluble form of the receptor (sR4) resulted in less severe CV and SK in WT infected animals. These observations could mean that targeting R4 could represent a useful therapeutic tool to counteract corneal angiogenesis and help control the severity of SK.

Several years ago CV was demonstrated to be a critical step in the pathogenesis of the blinding immunoinflammatory reaction to HSV-1 infection in the eye [17,18]. Multiple angiogenic factors were implicated as involved in causing the CV, with VEGF considered to be the principal angiogenic agonist [19,20]. Therapeutic management of CV which included targeting VEGF [6,7], its receptors [5] or some downstream signaling events [6,8,21], have shown various levels of efficacy. This report adds an alternative approach which represents the host’s own mechanism to limit the extent of pathological CV. Accordingly, R4 was initially shown to control the extent of endothelial migration in a non infection induced model of retinal and choroidal vascular diseases. These initial studies showed that animals unable to express R4 because of gene knockout developed heightened levels of angiogenesis [11]. We used a similar approach with an infection model of corneal disease and showed that animals lacking R4 expression developed heightened CV responses after HSV-1 infection as well as more severe SK. R4 expression is known to stabilize the vasculature by inhibiting processes stimulated by VEGF [22]. Given that R4 absence leads to leaky blood vessels that allow the escape of inflammatory substances [23,24], the elevated SK scores together with the increased cellular infiltration seen in R4 KO animals were anticipated consequences of increased neovascularization. In addition, since the elevated CV responses in R4 KO animals could largely be restored to levels observed in normal infected animals by the early local provision of the soluble form of R4, it further suggested that the extent of angiogenesis is regulated by this molecule. Of interest we could also show that the extent of CV in WT infected animals could be diminished when sR4 was administered to the subconjunctival site. Our results are in accordance with Sutching et al., who using the mouse subcutaneous sponge angiogenesis model, could reduce vessel development compared to control treatment [25]. However, in our case the administration of sR4 was not shown to be practical for reasons further discussed subsequently.

One issue that remains currently unclear is if R4 acts as a receptor or as a ligand. It was previously shown that Slit2 was the ligand of R4, by adding R4 expressing cells to supernant with myc-tagged Slit-2 transfected cells or in ELISA assays [26]. However, Sutching et al. results were the one which initially shed doubt as to whether Slit 2 is the actual ligand of R4 [25]. In fact, they could demonstrate by co-immunoprecipitation and Biocore binding assays that Slit2 does not bind to R4 [25]. If Slit2 binds to R4, the administration of sR4 would have sequestered Slit2, preventing it from interacting with Robo4 receptor and resulting in more angiogenesis. However, as mentioned before, in Sutching’s and our system the administration of sR4 to WT infected mice reduced the pathological angiogenesis compared to control animals. Later, Eichman’s group reconfirmed that Slit 2 was not the ligand of R4 and proposed that even though R4 is a receptor; its extracellular domains interacted with the vascular-specific axon guidance receptor uncoordinated 5 homolog β, UNC5β [27]. The latter interaction was confirmed by surface plasmon resonance. Moreover, using the corneal pocket assays with VEGF implants, it was shown that treatment of mice with anti-R4 and anti-UNC5β resulted in corneal hypervascularization [27]. Previously we also supported the hypothesis that Slit2 bind to R4 [21]. However in light of the current evidences, and the increased UNC5β transcripts detected after HSV-1 infection (data not shown) we favor the hypothesis in which R4 acts more as a ligand than a receptor.

Despite the successful results obtained when administered sR4 to reduced HSV-1 induced angiogenesis, for this approach to function, it was necessary to administer sR4 soon after infection. Delaying the therapy to later time points (even when given on day 6 pi. before notable CV becomes evident) had no therapeutic effect. Thus, targeting R4 does represent an approach that is effective but from a therapeutic perspective, administering sR4 would be inadequate in clinical settings. It will be necessary to explore ways of prolonging the administration of the reagent perhaps by using an expression vector system or focus instead on the signaling consequences of R4 manipulation. The signaling events have been shown to involve one or more SRC kinases, which in our study was shown to be elevated in R4 KO and less so in WT animals. Moreover, R4 KO animals demonstrated higher VEGF levels in corneas compared to infected WT mice. Further studies are also needed to compare the efficacy of targeting R4 itself or the events it triggers at various stages of infection for their effect on HSV-1 induced angiogenesis. In addition, since SK is a mutifactorial disease, combining sR4 with other approaches that target CV and/or inflammation would be another possible way to delay its administration.

In conclusion we have demonstrated that sR4 administration is a mean to enhance the antiangiogenic host feedback mechanism to reduce HSV-1 induced angiogenesis and subsequent immunopathology. Even though the mechanism is still under investigation, the results obtained suggest that R4 binds to an endothelial receptor to counteract VEGF signaling. Such receptor could be UNC5β. This is the first report that evaluates the relevance of sR4 in an infectious disease that involves pathological angiogenesis. Further research is underway to understand its mechanism and further develop a more practical therapy.
